# Predictive models for personalized precision medical intervention in spontaneous regression stages of cervical precancerous lesions

**DOI:** 10.1186/s12967-024-05417-y

**Published:** 2024-07-26

**Authors:** Simin He, Guiming Zhu, Ying Zhou, Boran Yang, Juping Wang, Zhaoxia Wang, Tong Wang

**Affiliations:** 1 Department of Health Statistics, School of Public Health, Shanxi Medical University, Taiyuan, 030001 China; 2 Key Laboratory of Coal Environmental Pathogenicity and Prevention, Shanxi Medical University, Ministry of Education, Taiyuan, 030001 China; 3 Department of Obstetrics and Gynecology, First Hospital of Shanxi Medical University, Taiyuan, 030001 China

**Keywords:** Predictive model, HPV infection, Cervical cancer, Machine learning, Pathological images

## Abstract

**Background:**

During the prolonged period from Human Papillomavirus (HPV) infection to cervical cancer development, Low-Grade Squamous Intraepithelial Lesion (LSIL) stage provides a critical opportunity for cervical cancer prevention, giving the high potential for reversal in this stage. However, there is few research and a lack of clear guidelines on appropriate intervention strategies at this stage, underscoring the need for real-time prognostic predictions and personalized treatments to promote lesion reversal.

**Methods:**

We have established a prospective cohort. Since 2018, we have been collecting clinical data and pathological images of HPV-infected patients, followed by tracking the progression of their cervical lesions. In constructing our predictive models, we applied logistic regression and six machine learning models, evaluating each model’s predictive performance using metrics such as the Area Under the Curve (AUC). We also employed the SHAP method for interpretative analysis of the prediction results. Additionally, the model identifies key factors influencing the progression of the lesions.

**Results:**

Model comparisons highlighted the superior performance of Random Forests (RF) and Support Vector Machines (SVM), both in clinical parameter and pathological image-based predictions. Notably, the RF model, which integrates pathological images and clinical multi-parameters, achieved the highest AUC of 0.866. Another significant finding was the substantial impact of sleep quality on the spontaneous clearance of HPV and regression of LSIL.

**Conclusions:**

In contrast to current cervical cancer prediction models, our model’s prognostic capabilities extend to the spontaneous regression stage of cervical cancer. This model aids clinicians in real-time monitoring of lesions and in developing personalized treatment or follow-up plans by assessing individual risk factors, thus fostering lesion spontaneous reversal and aiding in cervical cancer prevention and reduction.

**Graphical Abstract:**

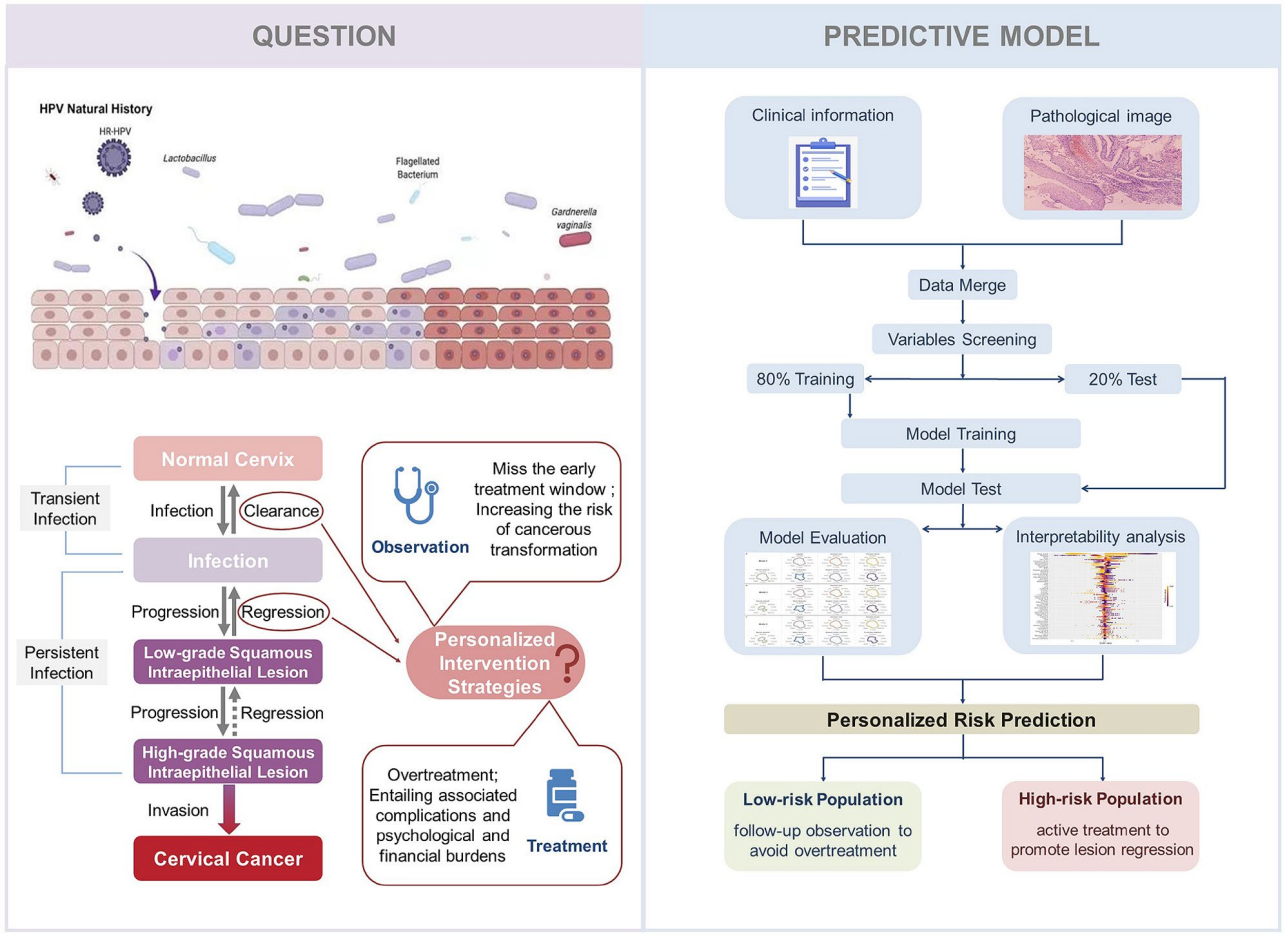

**Supplementary Information:**

The online version contains supplementary material available at 10.1186/s12967-024-05417-y.

## Introduction

Cervical cancer, a significant health threat to women, is primarily induced by Human Papillomavirus (HPV) infection. Over 80% of women experience at least one HPV infection in their lifetime [1], with the majority manifesting as asymptomatic and transient infections. However, in certain instances, the infection persists, potentially leading to mild cervical cell abnormalities known as Low-Grade Squamous Intraepithelial Lesion (LSIL) or Cervical Intraepithelial Neoplasia grade 1 (CIN1). This stage is generally reversible, with studies indicating that approximately 60–70% of LSIL cases spontaneously regress within one year, and this figure approaches approximately 90% within two years [2]. Should LSIL fail to resolve and HPV infection persists, the lesion may advance to High-Grade Squamous Intraepithelial Lesion (HSIL), encompassing Cervical Intraepithelial Neoplasia grades 2 and 3 (CIN2, CIN3). The probability of reversal at this stage is lower, yet spontaneous regression can still occur, particularly in younger women. Without appropriate treatment and management, HSIL will ultimately progress to cervical cancer. The prolonged period from HPV infection to cervical cancer development, highlighted by the high potential for reversal during the LSIL stage, provides a critical opportunity for cervical cancer prevention. Rational intervention at the LSIL stage can significantly enhance the prevention capabilities, effectively reducing the incidence of cervical cancer.

At present, intervention strategies for patients in the LSIL stage remain a contentious topic. According to the 2019 guidelines of the American Society for Colposcopy and Cervical Pathology (ASCCP), observation is the preferred approach for patients diagnosed with HPV-induced cervicitis and LSIL. Additionally, treatment is discretionary for high-risk HPV (hrHPV), a more significant proportion of high-grade lesions, and prolonged infection duration, based on patient preferences. With the implementation of screening policies and heightened health awareness, a significant number of women are detected with positive HPV infection or diagnosed at the LSIL stage during opportunistic screening. Due to the guidelines’ ambiguity regarding treatment, clinical practice currently relies more on empirical estimation of patient outcomes, subsequently guiding observation or pharmacological/surgical intervention. However, this intervention approach may lead to two adverse outcomes. For high-risk populations potentially progressing to HSIL, mere observation could miss the early treatment window, thereby increasing the risk of cancerous transformation [3, 4]. Conversely, aggressive interventions such as pharmacological, physical, and surgical treatments could lead to overtreatment, entailing associated complications [5, 6] and psychological [7] and financial burdens [8]. Moreover, current intervention plans do not fully consider individual differences. In resource-limited healthcare systems, treatments may consume resources that could be allocated to patients at higher risk.

Currently, intervention strategies and medical needs are progressively shifting towards novel treatment methods, precision medicine, early screening, and pre-cancer intervention strategies. The treatment and prevention of cervical cancer are trending towards personalized therapy, aiming to ensure oncological safety while minimizing the incidence rate. A recent comprehensive study outlined the global burden of cervical cancer, emphasizing the significance of early detection and intervention in improving prognosis, particularly in developing countries where rational intervention and treatment during early or pre-cancer stages are crucial in reducing the societal burden of cervical cancer [9]. Another review on the management of CIN stages also highlights the importance of CIN stages in cervical cancer prevention, stressing the necessity of predictive screening, appropriate intervention, and the application of precision medicine [4]. These findings underscore the pivotal role of the LSIL stage in the prevention, diagnosis, and treatment of cervical cancer. By accurately predicting the risk of lesion progression, these models assist in guiding personalized treatment decisions, optimizing resource allocation, and enhancing public awareness and participation in cervical cancer screening.

In early studies of cervical cancer prediction models, researchers primarily focused on predicting the survival or recurrence rates of cervical cancer patients [10, 11]. In recent years, attention has gradually shifted towards predicting the onset of cervical cancer, leading to the construction of numerous models for early risk prediction and screening [12]. Particularly, the application of machine learning methods has significantly improved the performance and accuracy of these models [13, 14]. With increasing recognition of the spontaneous regression potential of precancerous lesions, more researchers are acknowledging the importance of the CIN stage in cervical cancer prevention and control. Prediction models for this stage are gradually being developed. Austin et al. [15]. developed the Pittsburgh cervical cancer screening model based on 19 relevant variables, employing dynamic Bayesian methods to quantitatively estimate the risk of HSIL and carcinoma in situ in patients. Similarly, Charlton et al. [16]. utilized multivariate logistic regression to construct a model with HSIL and carcinoma in situ as the predicted outcomes, using basic clinical information to forecast the risk of cervical abnormality progression in patients with Atypical Squamous Cells of Undetermined Significance (ASCUS)/LSIL. However, the evaluation of this model revealed an Area Under the Curve (AUC) of only 0.63, indicating significant potential for enhancing its predictive performance. Koeneman et al. [17]. also applied multivariate logistic regression, basing their predictions on patient demographics and laboratory results to forecast spontaneous regression in CIN2 cases. The evaluation of their model similarly showed a relatively low AUC of 0.692. A recent study developed a predictive model using five variables: TCT results, HPV status, and the proportion of samples with acetowhite epithelium, abnormal blood vessels, and mosaicity [18]. The model demonstrated a good predictive performance (AUC = 0.851) for the prognosis of patients at the HSIL stage. However, as previously mentioned, the probability of regression for HSIL patients is lower compared to those at the LSIL stage. Although the model aims to predict the risk of cervical cancer in HSIL patients to reduce unnecessary surgeries and minimize side effects for low-risk patients, its effectiveness in promoting lesion regression and preventing cervical cancer may be somewhat limited.

The aforementioned models clearly demonstrate the effectiveness of predictive models in early identification of patients at high risk for cervical cancer. However, given the higher potential for reversal exhibited during the LSIL stage, models that advance their predictive endpoints to this earlier stage can facilitate the identification of high-risk LSIL cases, which is crucial for early intervention and the prevention of cervical cancer progression [18, 19]. Particularly for developing countries, such models can assist in optimizing screening protocols, focusing resources on high-risk populations, thereby reducing the risks of missed diagnoses and misdiagnoses. Furthermore, prediction models can assist physicians in developing personalized treatment plans for LSIL patients at varying levels of risk. For patients at lower risk, the model can guide the adoption of more conservative observation strategies, while those at higher risk may necessitate more proactive interventions. Additionally, these prediction models can inform public health strategies, aiding health departments in more effective resource allocation and providing data support to policymakers. This assistance is crucial in formulating more efficient cervical cancer screening and prevention programs, thereby enhancing the overall efficacy of cervical cancer prevention and treatment efforts.

In this study, our research primarily focuses on the spontaneously regressive stages of lesion development during the progression from HPV infection to cervical cancer, especially during the LSIL phase. Our objective is to construct a predictive model to assess the prognosis risk of patients with HPV infection or LSIL, and to identify key factors influencing the infection status. Our study is based on data from both clinical information and pathological images. The inclusion of pathological images, serving as the “gold standard” for determining lesion status, is crucial in ensuring the predictive accuracy of the model. Through the development of this predictive model, we aim to achieve real-time monitoring of the prognostic status of patients in the LSIL stage, assisting clinicians in making rational medical decisions and in devising personalized follow-up or treatment plans for patients. This approach is intended to effectively prevent disease progression and promote natural regression of the infection, thereby reducing the incidence and prevalence of cervical cancer.

## Methods

### Patients, selection criteria, and follow-up

In this prospective cohort study, we undertook the systematic collection of data from patients presenting with initial, persistent, or recurrent HPV infection, establishing an extensive prospective cohort initiated at the First Hospital of Shanxi Medical University in January 2018. The study adhered to strict exclusion criteria: (a) patients with concurrent mental disorders; (b) patients incapable of comprehending or completing the questionnaire due to speech or intellectual disabilities; (c) patients with concurrent life-threatening diseases. Following these inclusion and exclusion criteria, a total of 511 patients diagnosed with HPV infection were enrolled in the study. All patients participating in the study have signed informed consent forms. Enrolled subjects were subjected to regular follow-ups every three months for meticulous documentation of HPV infection status, transition to negativity, duration of recurrent and persistent infections, disease progression, and clinical outcomes. The follow-up mainly consisted through outpatient visits. For patients unable to attend the clinic follow-up, such as those not residing locally, we conducted follow-ups by telephone (*n* = 68). The phone call involved asking patients about their health status and collected detailed and complete records of their HPV tests and cervical lesion examination results from local tertiary hospitals within a three-month period. Additionally, to ensure consistency in the assessment of cervical lesion, all follow-up results, whether from outpatient visits or telephone follow-ups, were evaluated by the same professional physician.

### Clinical multi-parameters

All patients enrolled in the study were subjected to the Thinprep Cytologic Test (TCT) and HPV testing. Their infection status and specific HPV types were accurately determined based on the results of these diagnostic assessments. The TCT and HPV testing were performed during the patient’s non-menstrual period, with cell samples being scraped from the cervix. For the TCT, the collected cell samples were processed using a liquid-based cytology processor (ThinPrep 2000) to create thin-layer cell smears. The cell smears were then stained with Papanicolaou stain, and two cytopathology experts independently examined the smears under a microscope. For the HPV testing, the cervical samples were collected using the ThinPrep PreservCyt Solution to preserve the integrity of the nucleic acids within the cells. HPV testing utilizes Fully Automatic Nucleic Acid Hybridization Detector (Yaneng BIO YN-HR96) to detect the presence, viral load and type of HPV.

Informed by existing studies [3, 20, 21], we selected pertinent factors associated with cervical cancer and HPV infection to construct our questionnaire. The questionnaire comprehensively included sections on demographic data, socioeconomic status, family medical history, personal cervical disease history, sexual and reproductive history, health risk behaviors, anxiety and mental health, and sleep status. Among them, exercise, sleep quality, anxiety and mental health were assessed using standardized scales, specifically the IPAQ (International Physical Activity Questionnaire), PSQI (Pittsburgh Sleep Quality Index), SRSS (Self-Rating Scale of Sleep), and SAS (Self-Rating Anxiety Scale). The remaining variables were obtained through patient statements or self-reporting. For patients with incomplete questionnaires in the survey (*n* = 43), missing data were supplemented through timely telephone interviews with those patients to ensure the integrity and authenticity of the data. The details regarding the missing data in the questionnaire survey can be found in Supplementary Table S1.

Patient clinical data was entered using EpiData software (version 3.1), with the entry process independently completed by two trained operators and subjected to consistency verification. The clinical data of the patients were statistically described and compared between groups. Following the normality test, quantitative variables with a normal distribution were described using $\overline X {\rm{ \pm S}}$, and group comparisons were conducted using the independent sample *t*-test. Categorical variables were described using percentage, and group comparisons were performed using the *χ*^*2*^ test. The significance level was set at *α*<0.05.

### Pathological image acquisition and image feature extraction

Pathological biopsy is acknowledged as the gold standard for evaluating the extent of cervical intraepithelial lesions. In this study, pathological sections were obtained from patients during their nonmenstrual phase. The acquired samples were processed with hematoxylin-eosin staining and subsequently underwent pathological examination. High-resolution images of the pathological sections were captured using an image acquisition system (Axio Scope.A1) at a magnification of 100x (resolution of 2048 × 1536 pixels). Each sections was labeled by two experienced pathologists independently.

The analysis of the pathological images involved color feature extraction and texture feature extraction, tailored to the specific properties of the pathological tissue sections. Color feature extraction in this study encompassed three distinct color features: RGB features [22], HSV features [23], and Lab features [22]. The extraction of color features from pathological images was based on the color histogram. This involved grouping the pixels in the image by color and then counting the number of pixels within each color group to generate a color histogram, which effectively represents the color distribution characteristics of the image. The variables extracted included the mean and standard deviation for each channel of R, G, B, H, S, V, L, a, and b, in addition to the overall mean and variance. Texture feature extraction incorporated two texture attributes: the Grey Level Co-occurrence Matrix (GLCM) [24] and Gabor texture features [25]. To ensure precision and classification accuracy in texture feature extraction, GLCM contrast, energy, and correlation features were extracted at angles of 0, 45, 90, and 135 degrees, respectively. The process of image feature extraction was carried out utilizing the OpenCV library within the Python 3.7.0 environment. After extraction, these image features were integrated with clinical data to form a unified dataset, enabling further analysis and model development.

### Screening of variables

The predictive efficacy of a model is considerably influenced by the variables it incorporates. Factors such as the number of variables, inter-variable correlations, and the inclusion of critical variables significantly affect the accuracy and efficiency of the prediction model. Hence, the process of variable selection is crucial in the construction of an effective prediction model. In our study, we initially conducted a test for multicollinearity among the variables. Specifically, we assessed the presence of multicollinearity using the Corrected Generalized Variance Inflation Factor (CGVIF) [26], establishing a threshold where a CGVIF less than 5 indicates an acceptable level of multicollinearity. Subsequently, we employed the Boruta method [27] and the biosigner method [28] within the framework of the Random Forest (RF) variable importance scoring approach for variable screening. The set of influential factors to be included in the prediction model was determined based on the outcomes of this variable screening process.

The results of the biosigner method and Boruta method can be influenced by the quality and size of the dataset. Specifically, when the dataset is either too small or of suboptimal quality, the variable screening results might be inaccurate. Concurrently, the selection of an excessive number of variables by these methods can potentially lead to model overfitting. To mitigate these issues, we conducted an additional 500 iterations of 10-fold cross-validation within RF error rate. Ultimately, the optimal set of variables to be incorporated into the model was determined in consultation with the clinical chief physician, ensuring relevance and practical applicability.

### Model settings

In this study, we developed three distinct model scenarios: Model 1, which incorporates only clinical feature data; Model 2, comprising solely pathological image feature; and Model 3, a combination of both clinical feature data and pathological image feature data. For the modeling approach, we selected the logistic regression along with six widely utilized machine learning algorithms: Decision Tree (DT), RF, Naive Bayesian (NB), Support Vector Machine (SVM), K-Nearest Neighbor (KNN), and Neural Network (NN). The use of logistic regression, is due to its well-established application in binary outcome prediction, ease of interpretation, which are highly informative in clinical contexts. The application of multiple machine learning methods is based on their distinct advantages and capabilities in handling different types of data: DT offers interpretability and simplicity in visualizing decision paths; RF is known for its robustness to overfitting and ability to handle high-dimensional data effectively; SVM is effective in high-dimensional spaces and in cases where the number of dimensions exceeds the number of samples; KNN is simple to implement and understand, with effectiveness in local decision boundaries; NB is efficient and effective with small datasets and assumes independence between predictors, which can simplify the modeling process and NN are powerful in capturing complex non-linear relationships within the data.

### Model training and evaluation

**Fig. 1:**
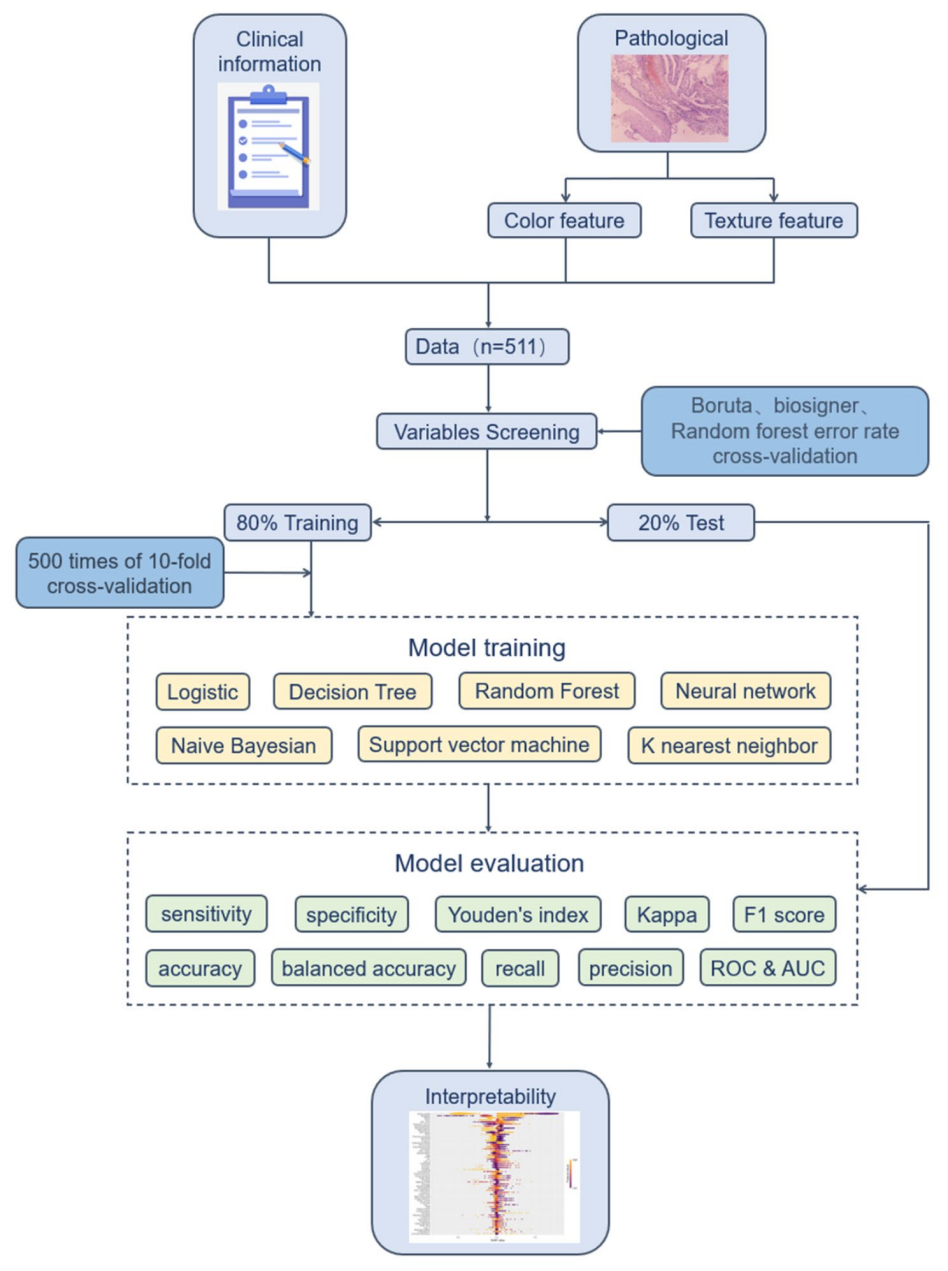
The flow chart of model construction

The dataset in our study was randomly divided into training and testing sets at a ratio of 8:2, and hyperparameter tuning was conducted using Grid Search Cross-Validation (GridSearchCV). To ascertain the optimal model, we performed 500 iterations of 10-fold cross-validation. The model evaluation metrics in our study encompassed a comprehensive range of indicators: sensitivity, specificity, Youden’s index, accuracy, balanced accuracy, precision, recall, Kappa coefficient, F1 score, Receiver Operating Characteristic curve (ROC), and AUC. Figure 1 depicts the detailed process of model construction.

### Interpretability analysis

To illuminate the significance of different features within our model, we employed Shapley Additive Explanations (SHAP), a post-hoc explanatory tool designed to decipher ‘black-box’ models using the Shapley value, a concept rooted in game theory. This approach quantifies the contribution of each feature to the model’s predictions, thereby enabling a detailed understanding of the impact of individual variables [29]. SHAP conceptualizes the Shapley value as a linear model where feature contributions are additive, transforming the model’s output into a sum of values attributed to each feature. It not only highlights the importance of each feature within the model but also delineates the directionality of their influence on the model’s predictive decision-making process.

## Results

### Description of clinical data

The study encompassed a total of 511 patients. Following the follow-up period, 244 patients experienced HPV clearance, while 267 continued to exhibit persistent HPV infection. We conducted a statistical analysis of the clinical data from all 511 participants, comparing characteristics between the HPV clearance group and the persistent infection group. The findings are presented in Table 1.

**Table 1:** Statistic description of clinical data

Influencing factors	Overall (*n* = 511)	persistent infection group (*n* = 267)	HPV clearance group (*n* = 244)	Statistics	*P* value
**Demographics**					
**Age (years)**	46.04 ± 10.89	47.33 ± 10.54	44.62 ± 11.10	2.83	0.005**
**Marriage status**				2.64	0.267
Married	484(94.72%)	256(95.88%)	228(93.44%)		
Divorced/separated/widowed	8(1.57%)	2(0.75%)	6(2.46%)		
Single	19(3.72%)	9(3.37%)	10(4.10%)		
**Educational level**				2.77	0.597
Bachelor’s degree or above	107(20.94%)	54(20.22%)	53(21.72%)		
Junior college and senior high school	147(28.77%)	72(26.97%)	75(30.74%)		
Junior high school	159(31.12%)	85(31.84%)	74(30.33)		
Primary school	77(15.07%)	46(17.23%)	31(12.70%)		
Illiteracy or little literacy	21(4.11%)	10(3.75%)	11(4.51%)		
**Socioeconomic status**					
**Type of occupation**				2.87	0.603
College students	1(0.2%)	1(0.37%)	0(0.00%)		
Employee	180(35.23%)	89(33.33%)	91(37.30%)		
Freelance work	63(12.33%)	30(11.24%)	33(13.52%)		
Unemployed	216(42.27%)	119(44.57%)	97(39.75%)		
Retiree	51(9.98%)	28(10.49%)	23(9.43%)		
**Main types of work**				0.78	0.676
Heavy physical labor	106(20.74%)	53(19.85%)	53(21.72%)		
Light physical labor	262(51.27%)	135(50.56%)	127(52.05%)		
Intellectual work	143(27.98%)	79(29.59%)	64(26.23%)		
**Main living area**				1.42	0.234
Rural	181(35.42%)	101(37.83%)	80(32.79%)		
City	330(64.58%)	166(62.17%)	164(67.21%)		
**Lifestyle and health risk behaviors**					
**Eating habits**				0.28	0.870
Vegetarian diet	87(17.06%)	44(16.48%)	43(17.70%)		
Vegetarian preference	288(56.47%)	150(56.18%)	138(56.79%)		
Meat preference	136( 26.47%)	73(27.34%)	63(25.51%)		
**Smoking**				0.0001	0.990
No	490(95.89%)	256(95.88%)	234(95.90%)		
Yes	21(4.11%)	11(4.12%)	10(4.10%)		
**Passive smoking**				1.28	0.259
No	261(51.08%)	130(48.69%)	131(53.69%)		
Yes	250(48.92%)	137(51.31%)	113(46.31%)		
**Drinking**				1.57	0.210
No	481(94.13%)	248(92.88%)	233(95.49%)		
Yes	30(5.87%)	19(7.12%)	11(4.51%)		
**Exercise (IPAQ)**				5.83	0.054
Low-intensity exercise	173(33.86%)	78(29.21%)	95(38.93%)		
Medium intensity exercise	314(61.45%)	174(65.17%)	140(57.38%)		
High-intensity exercise	24(4.70%)	15(5.62%)	9(3.69%)		
**Sexual**					
**Masturbation**				2.20	0.332
Never	263(51.47%)	138(51.69%)	125(51.23%)		
Once	207(40.51%)	112(41.95%)	95(38.93%)		
Always	41(8.02%)	17(6.37%)	24(9.84%)		
**Contraceptive methods**				3.74	0.439
Condom	76(14.87%)	36(13.48%)	40(16.39%)		
Oral contraceptives	8(1.57%)	3(1.12%)	5(2.05%)		
Intrauterine device	74(14.48%)	34(12.73%)	40(16.39%)		
No contraception	343(67.12%)	188(70.41%)	155(63.52%)		
Sterilization	10(1.96%)	6(2.25%)	4(1.64%)		
**Sexual hygiene**				1.24	0.539
Frequently clean the vagina	130(25.44%)	64(23.97%)	66(27.05%)		
Occasionally clean the vagina	234(45.79%)	121(45.32%)	113(46.31%)		
Never clean the vagina	147(28.77%)	82(30.71%)	65(26.64%)		
**Number of sexual partners**				3.43	0.180
1	357(69.86%)	179(67.0%)	178(49.9%)		
2	136(26.61%)	80(30.0%)	56(41.2%)		
3 or more	18(3.52%)	8(3.0%)	10(4.1%)		
**Age of first sex (years)**	23.22 ± 3.83	22.98 ± 3.74	23.49 ± 3.91	1.51	0.133
**Frequency of sex (times/month)**	5.30 ± 4.09	4.674 ± 3.98	5.988 ± 4.09	3.67	< 0.001**
**Times of pregnancy**	3.098 ± 1.624	3.27 ± 1.60	2.91 ± 1.634	2.57	0.010*
**Times of childbirth**	1.857 ± 1.172	2.01 ± 1.214	1.69 ± 1.104	3.06	0.002**
**Sleep**					
**PSQI**				4.97	0.083
Good sleep quality (0–5)	139(27.20%)	65(24.34%)	74(30.33%)		
Not bad sleep quality (6–10)	304(59.49%)	159(59.55%)	145(59.43%)		
Average sleep quality (11–15)	68(13.31%)	43(16.10%)	25(10.25%)		
Poor sleep quality (16–21)	0(0.00%)	0(0.00%)	0(0.00%)		
**SRSS**				25.62	< 0.001**
Sleep well ($\le$23)	145(28.38%)	50(18.73%)	95(38.93%)		
Sleep poor (> 23)	366(71.62%)	217(81.27%)	149(61.07%)		
**Mental health and anxiety**					
**Status of mental health**				0.18	0.675
Good mental state	346(67.71%)	183(68.54%)	163(66.80%)		
Poor mental state	165(32.29%)	84(31.46%)	81(33.20%)		
**SAS**				14.24	0.002**
No anxiety (< 50)	168(32.88%)	71(26.59%)	97(39.75%)		
Mild anxiety (50–59)	204(39.92%)	120(44.94%)	84(34.43%)		
Moderate anxiety (60–69)	131(25.64%)	69(25.84%)	62(25.41%)		
Severe anxiety (> 69)	8(1.57%)	7(2.62%)	1(0.41%)		
**Status of HPV infection**					
**HPV infection duration (days)**	459.99 ± 366.28	482.42 ± 403.81	435.45 ± 319.25	1.45	0.148
**HPV infection strain**				2.55	0.279
16+/18+	241(47.16%)	133(49.8%)	108(44.3%)		
31+/45+/52+/53+/58+	147(28.77%)	77(28.8%)	70(28.7%)		
Others	123(24.07%)	57(21.3%)	66(27.0%)		

*Notations* *p < 0.05; **p < 0.01

*Abbreviation* IPAQ: The International Physical Activity Questionnaire; PSQI: Pittsburgh Sleep Quality Index; SRSS: Self-Rating Scale of Sleep; SAS: Self-Rating Anxiety Scale

The results revealed significant differences between the two groups in several aspects, including age, frequency of sexual activity, pregnancy history, reproductive history, sleep quality (as measured by the Self-Rating Scale of Sleep, SRSS), and anxiety levels (as assessed by the Self-Rating Anxiety Scale, SAS). Specifically, the average age in the persistent infection group (47.33 ± 10.54 years) was higher compared to the HPV clearance group (44.62 ± 11.10 years). The frequency of sexual activity in the persistent infection group (4.674 ± 3.98 times/month) was lower than that in the HPV clearance group (5.988 ± 4.09 times/month). Regarding pregnancy and childbirth history, the HPV clearance group had fewer instances compared to the persistent infection group. In terms of sleep and anxiety status, the patients in the HPV clearance group reported better sleep quality and lower levels of anxiety compared to those in the persistent infection group.

### Variable screening

**Fig. 2:**
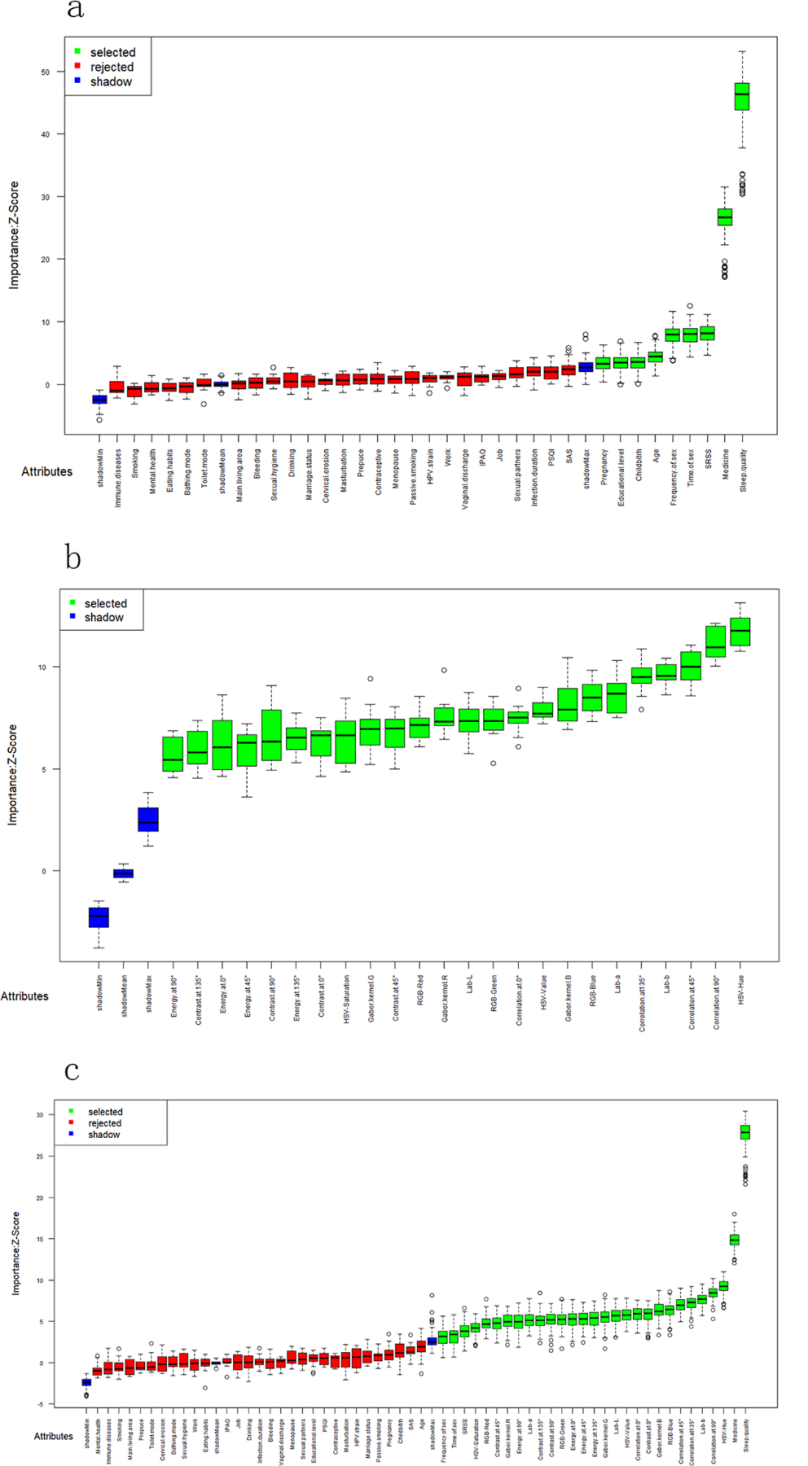
Boruta method variable screening results: (**a**) Boruta method variable screening results of model 1; (**b**) Boruta method variable screening results of model 2; (**c**) Boruta method variable screening results of model 3

**Fig. 3:**
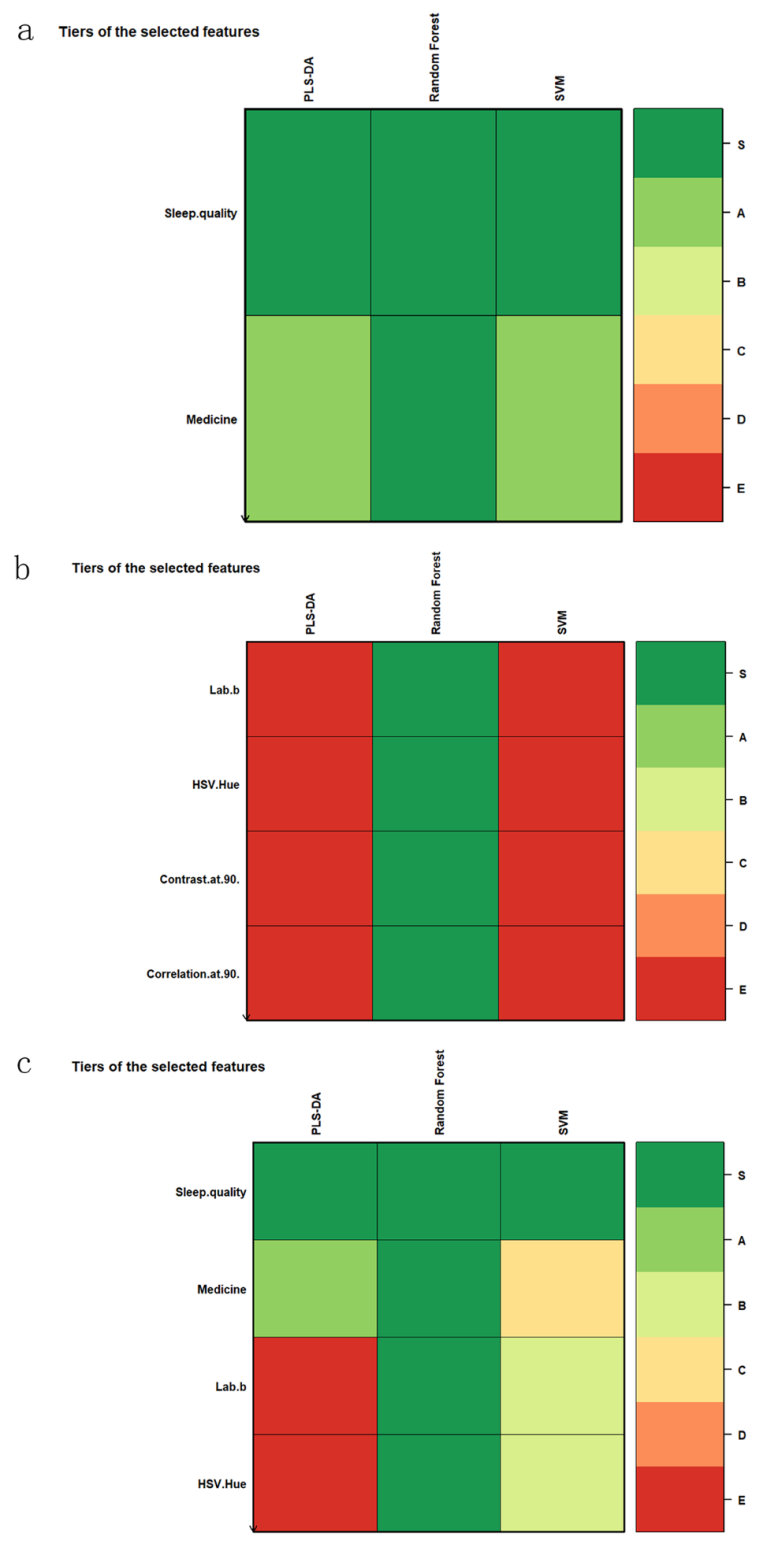
biogenser method variable screening results: (**a**) biogenser method variable screening results of model 1; (**b**) biogenser method variable screening results of model 2; (**c**) biogenser method variable screening results of model 3

The results shown that none variables have CGVIF exceeded 5, indicating the absence of collinear variables (Supplementary Table S2). Consequently, we utilized the biosigner and Boruta methods within the RF algorithm for variable importance scoring to conduct variable screening in all variables. The selection of influencing factors for the prediction model was based on the outcomes of the variable screening process. In Model 1, which focused solely on clinical variables, the Boruta method identified nine influential factors, including sleep quality. When pathological image features were incorporated in Model 3, the Boruta method expanded its selection to 29 variables, comprising five clinical variables and 24 image features. In contrast, the biosigner method, known for selecting the minimal optimal set of variables as features [28], identified only sleep quality as the influential factor in both Model 1 and Model 3. The detailed results of this variable selection process are depicted in Figs. 2 and 3.

**Fig. 4:**
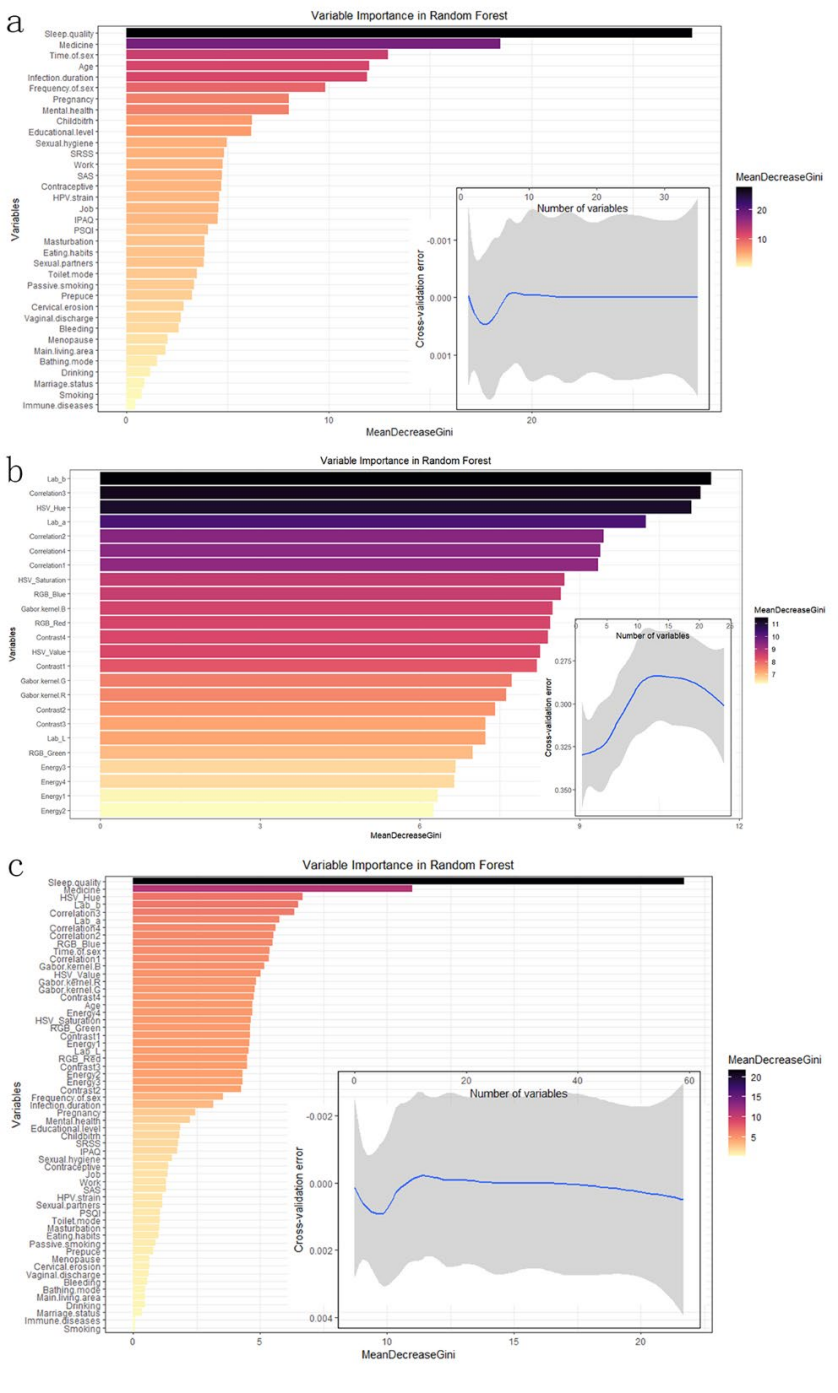
Cross-validation error rate results: (**a**) Cross-validation error rate results of model 1; (**b**) Cross-validation error rate results of model 2; (**c**) Cross-validation error rate results of model 3

Given that the variable screening outcomes from the biosigner and Boruta methods can be affected by the size and quality of the dataset, we executed 500 iterations of 10-fold cross-validation for evaluating the RF error rate. The results are presented in Fig. 4. Ultimately, guided by clinicians, we determined the optimal set of variables to be included in the model. The model was thus constructed with a total of 31 feature variables, which include 7 clinical features, color features from RGB, HSV, and Lab spectra, GLCM contrast, energy, correlation features, as well as Gabor texture features. Table 2 delineates this optimal feature set of the model.

**Table 2:** Feature set included in the model

Optimal feature set	Variable type	Variable notes
**Clinical variable**		
Age	Quantitative variable	years
Main types of work	Categorical variables	1 = heavy physical labor; 2 = light physical labor; 3 = intellectual work
Age of first sex	Quantitative variable	years
Frequency of sex	Quantitative variable	Times/month
SRSS	Categorical variables	1 = sleep well ($\le$23); 2=sleep poor (>23)
Status of mental health	Categorical variables	1 = good mental state; 2 = poor mental state
HPV infection duration	Quantitative variable	Days
**Color features**		
RGB-Red	Quantitative variable	[0, 255]
RGB-Green	Quantitative variable	[0, 255]
RGB-Blue	Quantitative variable	[0, 255]
HSV-Hue	Quantitative variable	[0, 360]
HSV-Saturation	Quantitative variable	[0, 1]
HSV-Value	Quantitative variable	[0, 1]
Lab-Lightness	Quantitative variable	[0, 100]
Lab-a (Green-Red)	Quantitative variable	[-128, 127]
Lab-b (Blue-Yellow)	Quantitative variable	[-128, 127]
**Texture features**		
GLCM contrast at 0°	Quantitative variable	[0, (256-1)^2^]
GLCM contrast at 45°	Quantitative variable	[0, (256-1)^2^]
GLCM contrast at 90°	Quantitative variable	[0, (256-1)^2^]
GLCM contrast at 135°	Quantitative variable	[0, (256-1)^2^]
GLCM energy at 0°	Quantitative variable	[0, 1]
GLCM energy at 45°	Quantitative variable	[0, 1]
GLCM energy at 90°	Quantitative variable	[0, 1]
GLCM energy at 135°	Quantitative variable	[0, 1]
GLCM Correlation at 0°	Quantitative variable	[-1, 1]
GLCM Correlation at 45°	Quantitative variable	[-1, 1]
GLCM Correlation at 90°	Quantitative variable	[-1, 1]
GLCM Correlation at 135°	Quantitative variable	[-1, 1]
Gabor kernel mean for images R	Quantitative variable	[0, 255]
Gabor kernel mean for images G	Quantitative variable	[0, 255]
Gabor kernel mean for images B	Quantitative variable	[0, 255]

*Abbreviation* SRSS: Self-Rating Scale of Sleep; GLCM: Grey Level Co-occurrence Matrix

### Model evaluation and comparison

**Fig. 5:**
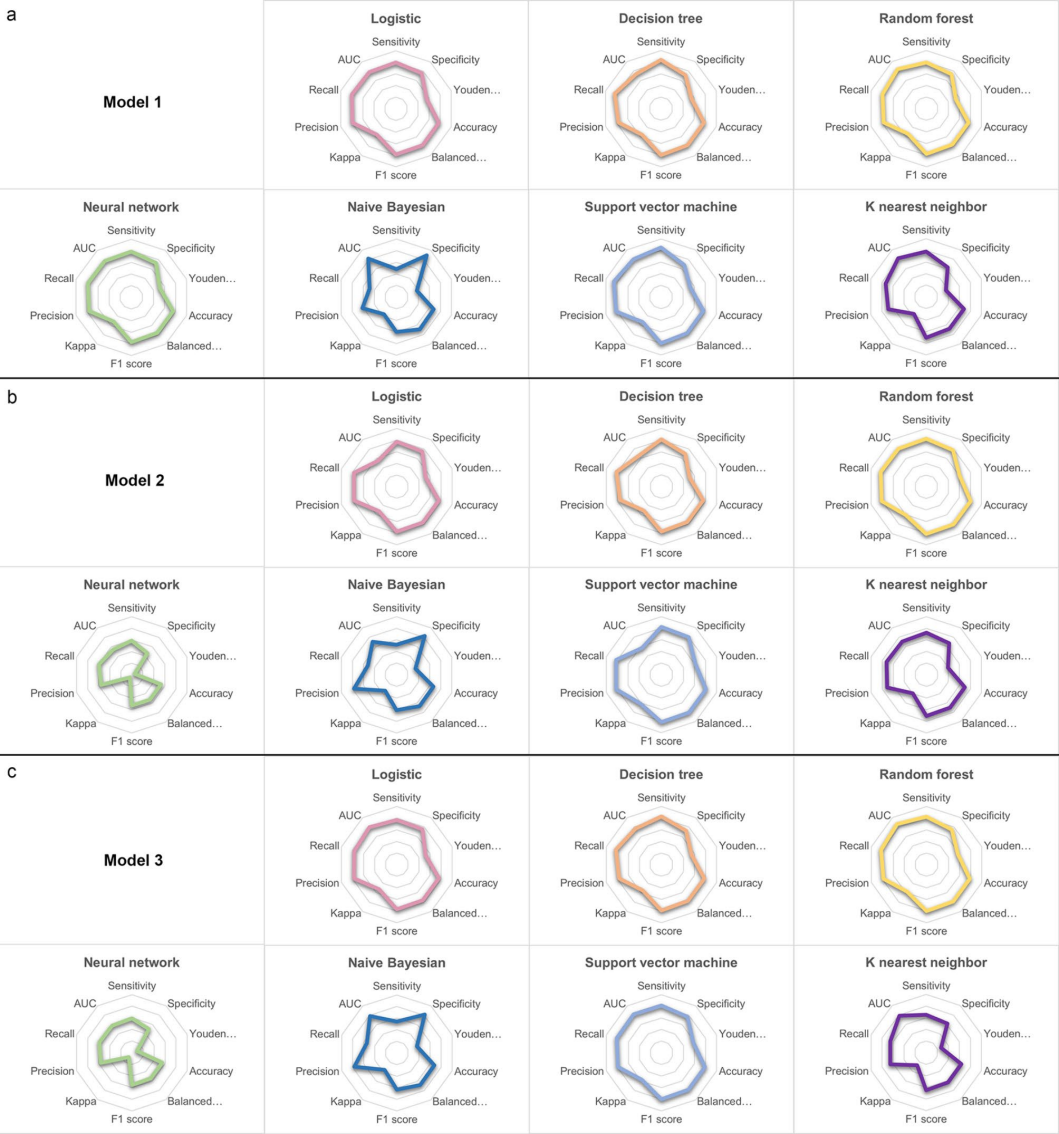
Model evaluation results: (**a**) Model evaluation results of model 1; (**b**) Model evaluation results of model 2; (**c**) Model evaluation results of model 3

In Model 1, which incorporated only clinical data, Logistic Regression and DT demonstrated superior performance across various comprehensive evaluation metrics. Logistic Regression achieved the highest Youden’s Index (0.555), balanced accuracy (0.778), and Kappa coefficient (0.555). Meanwhile, DT excelled with the highest accuracy (0.779), F1 score (0.798), and a Kappa coefficient equal to that of Logistic Regression (0.555). Additionally, the RF method presented the highest AUC value (0.841). In Model 2, which utilized only pathological image data, RF and SVM surpassed other methods. SVM recorded the highest values in Youden’s Index (0.617), accuracy (0.809), balanced accuracy (0.809), F1 score (0.817), Kappa coefficient (0.617), and precision (0.820). RF, on the other hand, showed the highest sensitivity (0.826), recall (0.826), and AUC (0.812). For the comprehensive Model 3, integrating both clinical and imaging data, RF and SVM again displayed better predictive performance. In this model, SVM had the highest scores in Youden’s Index (0.575), accuracy (0.789), balanced accuracy (0.788), Kappa coefficient (0.576), and precision (0.790). RF led with the highest F1 score (0.800) and AUC (0.866). The results are depicted in Fig. 5. Comparing all three models, Model 3 generally outperformed Models 1 and 2, especially with SVM and RF, indicating the best predictive capability among the three. Model 2 exhibited the weakest predictive performance in comparison to the other models. The detailed results of model evaluation are outlined in Table 3.

**Table 3:** Model evaluation results

Model		Sensitivity	Specificity	Youden index	Accuracy	Balanced accuracy	F1 score	Kappa	Precision	Recall	AUC
Model 1											
	Logistic	0.798	0.757	0.555	0.778	0.778	0.789	0.555	0.786	0.798	0.780
	DT	0.838	0.715	0.552	0.779	0.777	0.798	0.555	0.768	0.838	0.733
	RF	0.795	0.737	0.533	0.768	0.766	0.780	0.534	0.772	0.795	0.841
	NN	0.788	0.729	0.517	0.760	0.758	0.773	0.517	0.766	0.788	0.774
	NB	0.481	0.888	0.369	0.676	0.685	0.599	0.363	0.831	0.481	0.820
	SVM	0.851	0.682	0.533	0.770	0.767	0.794	0.537	0.750	0.851	0.804
	KNN	0.731	0.630	0.361	0.683	0.680	0.705	0.362	0.687	0.731	0.820
Model 2											
	Logistic	0.775	0.748	0.523	0.762	0.762	0.772	0.523	0.775	0.775	0.550
	DT	0.815	0.694	0.509	0.757	0.755	0.777	0.512	0.749	0.815	0.661
	RF	0.826	0.779	0.605	0.803	0.802	0.814	0.605	0.808	0.826	0.812
	NN	0.591	0.460	0.051	0.528	0.526	0.529	0.051	0.558	0.591	0.540
	NB	0.517	0.825	0.343	0.665	0.671	0.611	0.338	0.774	0.517	0.704
	SVM	0.820	0.797	0.617	0.809	0.809	0.817	0.617	0.820	0.820	0.568
	KNN	0.722	0.672	0.394	0.698	0.697	0.713	0.394	0.710	0.722	0.704
Model 3											
	Logistic	0.768	0.755	0.523	0.762	0.762	0.768	0.523	0.775	0.768	0.798
	DT	0.828	0.719	0.547	0.775	0.774	0.792	0.549	0.767	0.828	0.766
	RF	0.826	0.745	0.571	0.787	0.786	0.800	0.572	0.781	0.826	0.866
	NN	0.591	0.501	0.093	0.548	0.546	0.557	0.093	0.562	0.591	0.577
	NB	0.543	0.819	0.363	0.677	0.681	0.631	0.359	0.770	0.543	0.784
	SVM	0.813	0.763	0.575	0.789	0.788	0.798	0.576	0.790	0.812	0.817
	KNN	0.649	0.619	0.269	0.635	0.634	0.646	0.269	0.649	0.649	0.784

*Abbreviation* AUC: the area under the receiver operating characteristic curve; DT: decision tree; RF: random forest; NN: neural network; NB: naive Bayesian; SVM: support vector machine; KNN: K nearest neighbor

### Results of interpretability analysis

**Fig. 6:**
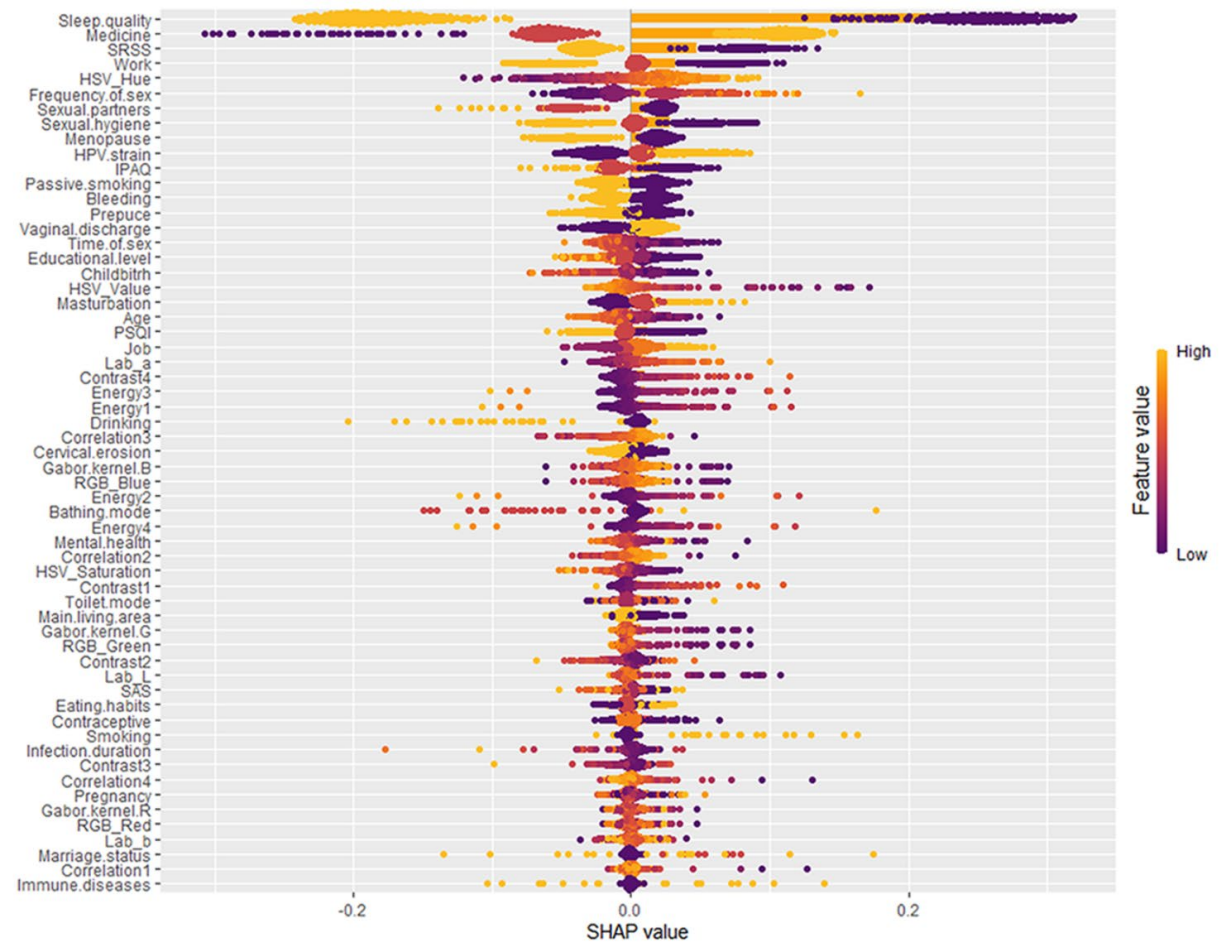
Overall model interpretability analysis

We utilized SHAP for the interpretability analysis of our predictive model, and assign importance rankings to the variables included in the model. The results, depicted in Fig. 6, are based on interpretability analysis conducted across the entire sample population. Each patient’s attribution results for the importance of each variable feature are represented by colored dots, where orange signifies high-risk values and purple signifies low-risk values. On an entire sample scale, considering the multitude of factors influencing HPV clearance in patients, the top five factors in terms of importance were identified as sleep quality, medication usage, short-term sleep problems, type of work, and sexual frequency. Among these, sleep quality is particularly noteworthy, exhibiting a significant impact on HPV clearance. This insight underscores the critical role of sleep quality in the prognosis of HPV infection outcomes.

**Fig. 7:**
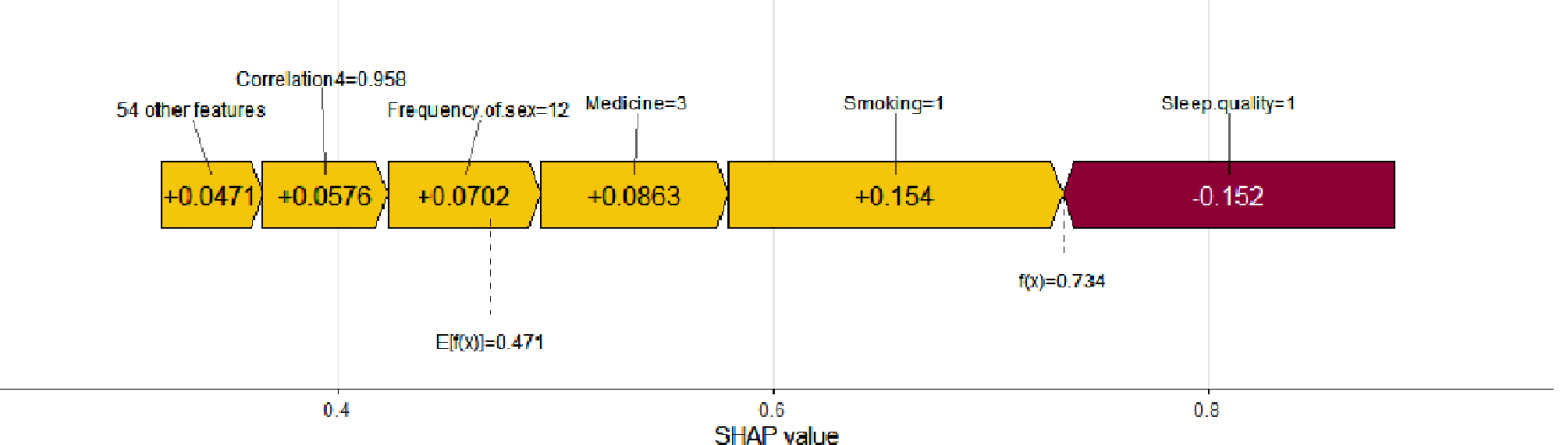
Model interpretability analysis of individuals

In clinical practice, SHAP interpretability analysis for individual patients can help clinicians gain a more intuitive understanding of the patient’s prognosis and the main influencing factors. This, in turn, enables them to provide personalized medical intervention strategies for the patient. For example, Fig. 7 displays the results of the SHAP explanatory analysis for an individual patient. In this case, the model computed a SHAP value of 0.471, with a corresponding prediction score for the patient being 0.734. This indicates a higher likelihood of this patient to undergo spontaneous HPV conversion or regression of lesions. Among the numerous factors that may influence spontaneous HPV clearance, smoking, sleep quality, medication, and sexual frequency exhibit the greatest explanatory power.

## Discussion

HPV has been identified as a significant factor contributing to the development of cervical cancer. Indeed, HPV-driven cancer is a relatively rare occurrence, as most infections are transient and can be spontaneously cleared by the host immune system. In cases of persistent HPV infection, it may take decades to progress to cervical cancer. This extended temporal window provides a golden opportunity for clinical prevention and early intervention. This study focuses on the stages of HPV infection with a higher potential for spontaneous regression during the progression to cervical cancer, particularly the stages of HPV infection accompanied by chronic inflammation and LSIL. In response to the current ambiguous treatment scenario at this stage and its pivotal role in cervical cancer prevention, we have developed a personalized precision medicine prediction model. This model not only predicts the progression of the disease based on the patient’s current condition and individual factors but also identifies the pivotal factors influencing the disease during its development. The evaluation of the model demonstrates that the predictive model we developed exhibits commendable performance in prediction capabilities, with an AUC of 0.866. Additionally, the identification of key influencing factors within the study confirmed the significant role of sleep quality in the spontaneous regression of LSIL and the spontaneous clearance of HPV infections.

In the preceding studies, researchers have recognized the significant role of the HSIL stage in the development of cervical cancer. Austin et al. [15]. employed 19 relevant variables, including HPV vaccination data, Papanicolaou test results, high-risk HPV infection, operation data, and histopathological results, to construct a Pittsburgh Cervical Cancer Screening model. Utilizing dynamic Bayesian methods, the researchers quantitatively assessed the risks associated with HSIL and adenocarcinoma in situ. However, this model primarily relies on molecular markers and laboratory indicators to predict the occurrence of cervical cancer. In practical clinical applications, its use may be limited by the accessibility of variable information. Following this, Charlton et al. [16] also focused on predicting the occurrence of HSIL and adenocarcinoma in situ, utilizing basic clinical information (age, smoking habits, number of sexual partners, pregnancies, immune status, etc.) to predict the risk of cervical abnormalities in ASCUS or LSIL patients. They employed multivariate logistic regression to construct the model, and the results indicated the predictive role of abnormal Papanicolaou test results in the further progression of cervical lesions. However, the model evaluation results showed an AUC of only 0.63, suggesting considerable room for improvement in predictive performance. In a similar vein, Koeneman et al. [17]. applied multivariate logistic regression to forecast the spontaneous regression in HSIL patients, based on demographic and laboratory data. The derived model, however, also exhibits a relatively low AUC of 0.692.

Our study presents two key enhancements building upon existing research. Firstly, our model advances the prediction outcome to the LSIL stage, given that this stage exhibits a higher potential for spontaneous regression. Consequently, timely detection and appropriate intervention at this stage hold greater significance and impact for the prevention of cervical cancer. With the assistance of this model, clinicians can monitor the lesion conditions of patients in real-time and, by evaluating individual risk factors, guide the development of personalized treatment or follow-up plans. For LSIL patients with a low risk of progression, this approach can avoid unnecessary treatments, thereby significantly reducing potential side effects and medical costs. Secondly, our predictive model integrates a multidimensional data including clinical information, laboratory test results, and pathological imaging. The inclusion of such a comprehensive range of variables significantly ensures the predictive efficacy of the model. The evaluation results also indicate that our model exhibits a substantial improvement in predictive performance compared to existing models. Additionally, in designing the model, we not only focused on ensuring its accuracy but also considered its clinical practicality. The variables included in our model are all accessible through routine diagnostic and treatment processes for LSIL patients or through simple questionnaires administered during regular clinical visits. This setup aims to ensure that the model can be easily integrated into existing clinical workflows while minimizing additional economic and medical burdens on patients and clinicians. However, the practical application of the model in clinical settings must also be comprehensively considered. Given that the optimal model in this study still includes some laboratory test indicators and pathological images, in relatively resource-limited situations where laboratory test indicators are unavailable, model1, which has a lower dependency on laboratory tests while maintaining relatively good predictive performance, can be considered significant. Conversely, when laboratory conditions permit, it is recommended to prioritize the use of model3, which integrates pathological images into the LSIL prediction model and offers better predictive performance compared to model1.

Another significant finding of our study is the identification of the role of sleep quality in the spontaneous clearance process following HPV infection. Both variable importance screenings and SHAP analyses of the predictive model highlight that enhanced sleep quality markedly facilitates the spontaneous clearance of HPV and the reversal of LSIL. This finding may be explicated from several perspectives: Firstly, sleep quality may influence the outcome of HPV infection by affecting immune function. The immune system plays a crucial role in clearing HPV and preventing the infection from progressing to more severe lesions. Poor sleep quality could weaken the immune system’s efficacy, thus potentially reducing the body’s ability to clear HPV infection [30–32]. Secondly, poor sleep quality may increase chronic inflammation in the body [33, 34], which is considered a significant factor in the development of cervical cancer [35, 36]. Therefore, sleep quality might affect HPV clearance and LSIL reversal through inflammatory responses. Thirdly, sleep quality can affect the balance of hormones such as estrogen and progesterone, which have been implicated in the course of HPV infection and the pathogenesis of cervical lesions [37–39]. Fourthly, sleep issues may increase psychological stress in patients. Research shows that psychological stress can prolong and exacerbate the duration and severity of HPV-related diseases [40–42]. Lastly, Sleep is involved in the regulation of cellular repair processes, including DNA repair. Inadequate sleep may impair the body’s ability to repair DNA damage in cervical cells caused by HPV, leading to increased risk of lesion progression [43, 44]. Similar research findings have been corroborated in other studies. Sims et al [45]. highlighted that individual aged over 65, insufficient sleep, disruption of the circadian rhythm, and chronic stress may contribute to stress-related insomnia in women, thereby elevating susceptibility to cervical-vaginal infections and the associated risk of cervical cancer. Garbarino et al. [46]. and Moscicki et al. [47]. independently validated the impact of sleep quality on cervical cancer, examining it through the lenses of microbiology and cytology, respectively. Additionally, Li et al. [38]. identified a correlation indicating that a diminished sleep quality score is associated with an elevated risk of cervical cancer, based on their prospective study within the Kailuan Cohort. This result provides novel perspectives for clinical interventions at this stage. In clinical work, integrating the assessment and management of sleep quality into the process of diagnosis and treatment can improve the quality of sleep of patients, which can better promote the reversal of lesions and spontaneous clearance of HPV, thereby effectively preventing cervical cancer.

With the rapid advancement of big data, machine learning methods have become a vital analysis method in emerging data science. In this study, we used six machine learning methods and logistic regression to develop a predictive model. Among those machine learning methods, RF stood out among them benefiting from the ability to process high-dimensional data and its capability to capture nonlinear relationships between features. RF enhances accuracy by constructing multiple DTs and to ensemble their predictions, thereby mitigating the risk of overfitting and augmenting the model’s generalization capacity. However, it is important to recognize that machine learning does not supplant traditional statistical analysis. Specifically, parametric models have the potential to outperform machine learning algorithms, especially when dealing with small datasets. This is also reflected in our study, where the predictive performance of the logistic regression model is better than that of machine learning algorithms such as NN and KNN. This may be due to the fact that as the complexity of the model increases, the fitting error of the training data decreases, which may lead to the problems of overfitting and generalization error [48]. Another important issue to consider in the application of machine learning models is the privacy of personal health data. In this study, the model was constructed with full attention to data privacy. In future applications, strict measures such as anonymization should be employed to ensure the protection of data privacy.

Several limitations should be considered. First, the models were developed based on the dataset derived from a single center. Due to the relatively limited focus on the LSIL stage and the availability of related datasets, external validation was not feasible within the scope of this study. We have planned to undertake this crucial step in future research. Despite these challenges, we have made efforts to ensure the generalizability of our predictive model. Firstly, our study cohort was sourced from a tertiary A-level hospital, with patients coming from a wide geographic area, which improves sample representativeness. Meanwhile, we performed extensive cross-validation to enhance the model’s robustness. In the future, we will further expand the scope of cohort studies while actively seeking partnerships for multicenter research. This will allow for more extensive external independent validation to confirm the model’s generalizability and robustness. Furthermore, this study focused on predicting disease progression following HPV infection based on pathological images and clinical features. While external environmental factors play a role in disease occurrence and development, the impact of genetics on disease progression cannot be overlooked. In subsequent studies, we plan to incorporate genetic influences into the model. Beyond genetics, the model can be enriched with epidemiological data, biomarker information, various omics data, and other characteristic data related to individual infection risk. These multi-omics data and broader data types can provide a more comprehensive view of the biological processes involved in the progression of LSIL, help identify new biomarkers, and elucidate complex interactions that might not be evident through single-omics analysis. Enhancing the model with these diverse data types can further improve the comprehensiveness of risk assessment and increase its practical value. This, in turn, provides clearer and more effective guidance for intervention strategies against cervical cancer and its precancerous lesions. Additionally, developing a dynamic model of lesion progression is an important research direction. Establishing such a model is crucial for understanding real-time disease progression in patients and adjusting medical intervention strategies accordingly. In the future, we will consider further developing dynamic models to enhance the disease risk assessment process. Our aim is to achieve dynamic evaluation of the entire disease progression, providing more effective tools for treatment intervention during the LSIL stage and cervical cancer prevention.

## Conclusion

This study specifically targeted crucial spontaneous regression stages in the progression from HPV infection to cervical cancer. The developed prognostic model for cervical precancerous lesions ensures high predictive performance and includes interpretability analysis. The model aids in individualized risk prediction for patients. Combined with existing clinical guidelines, this model can help clinicians gain a more intuitive understanding of a patient’s current disease progression status in clinical practice. The model’s predictions can assist in determining more personalized intervention strategies for patients. In comparison to existing cervical cancer prediction models, this study advances the predicted outcomes to the spontaneous regression stages in the disease development process. This innovation holds significant implications for enhancing comprehensive prevention capabilities, ultimately contributing to a reduction in the societal burden of cervical cancer.

### Electronic supplementary material

Below is the link to the electronic supplementary material.

Supplementary Material 1

## Data Availability

All data and code relevant to the study that are not in the article and supplementary material are available from the corresponding author on reasonable request.

## Abbreviations

ASCCP: American Society for Colposcopy and Cervical Pathology
ASCUS: Atypical squamous cell of undetermined significance
AUC: Area under the ROC curve
CIN: Cervical intraepithelial neoplasia
DT: Decision tree
GLCM: Grey Level Co-occurrence Matrix
HPV: Human papillomavirus
hrHPV: High-risk HPV
HSIL: High-grade squamous intraepithelial lesions
KNN: K nearest neighbor
LSIL: Low-grade squamous intraepithelial lesions
NB: Naive Bayesian
NN: Neural network
RF: Random forests
ROC: Receiver operating characteristic curve
SAS: Self-Rating Anxiety Scale
SHAP: Shapley additive explanations
SRSS: Self-Rating Scale of Sleep
SVM: Support vector machines
TCT: Thinprep cytologic testing
