# Supplementary material for: Predictive models for personalized precision medical intervention in spontaneous regression stages of cervical precancerous lesions
Source: J Transl Med. 2024 Jul 26;22:686. doi: 10.1186/s12967-024-05417-y (PMC11282852; doi:10.1186/s12967-024-05417-y)
Supplement: Supplementary file 1 — Supplementary Material 1 [file 12967_2024_5417_MOESM1_ESM.docx]

Table S1 Description of Missing Data in Questionnaire Survey

| Variable | n (percentage) |
| --- | --- |
| Marriage status | 4 (0.8%) |
| Educational level | 1 (0.2%) |
| Main living area | 8 (1.6%) |
| Eating habits | 3 (0.6%) |
| Smoking | 12 (2.3%) |
| Passive smoking | 12 (2.3%) |
| Drinking | 10 (2.0%) |
| Contraceptive methods | 5 (1.0%) |
| Times of pregnancy | 2 (0.4%) |
| Times of childbirth | 2 (0.4%) |

Table S2 Results of Variable Multicollinearity Test

| Variable | GVIF | CGVIF |
| --- | --- | --- |
| Age | 3.59 | 1.89 |
| Marriage status | 1.50 | 1.22 |
| Educational level | 2.31 | 1.52 |
| Type of occupation | 1.74 | 1.32 |
| Main types of work | 1.90 | 1.38 |
| Main living area | 2.12 | 1.46 |
| Eating habits | 1.47 | 1.10 |
| Smoking | 1.22 | 1.11 |
| Passive smoking | 1.12 | 1.06 |
| Drinking | 1.21 | 1.10 |
| Exercise (IPAQ) | 1.22 | 1.11 |
| Masturbation | 1.66 | 1.29 |
| Contraceptive methods | 1.30 | 1.14 |
| Sexual hygiene | 1.29 | 1.13 |
| Number of sexual partners | 1.47 | 1.21 |

| Age of first sex | 3.68 | 1.92 |
| --- | --- | --- |
| Frequency of sex | 2.41 | 1.16 |
| Times of pregnancy | 2.74 | 1.65 |
| Times of childbirth | 3.39 | 1.84 |
| Sleep quality (PSQI) | 1.34 | 1.08 |
| Sleep quality (SRSS) | 1.50 | 1.22 |
| Mental health | 1.23 | 1.11 |
| Anxiety (SAS) | 1.66 | 1.29 |
| HPV infection duration | 1.16 | 1.07 |
| HPV infection strain | 1.11 | 1.05 |

Abbreviation: GVIF: Generalized Variance Inflation Factor; CGVIF: Corrected Generalized Variance Inflation Factor; IPAQ: The International Physical Activity Questionnaire; PSQI: Pittsburgh Sleep Quality Index; SRSS: Self-Rating Scale of Sleep; SAS: Self-Rating Anxiety Scale.
